# Health Data Collection Before, During and After Emergencies and Disasters—The Result of the Kobe Expert Meeting

**DOI:** 10.3390/ijerph16050893

**Published:** 2019-03-12

**Authors:** Tatsuhiko Kubo, Alisa Yanasan, Teodoro Herbosa, Nilesh Buddh, Ferdinal Fernando, Ryoma Kayano

**Affiliations:** 1 Department of Environmental Epidemiology, Institute of Industrial Ecological Sciences, University of Occupational and Environmental Health, Kitakyushu 807-8555, Japan; 2 Ministry of Public Health, Nonthaburi 11000, Thailand; yanasan.a@gmail.com; 3 UP College of Medicine, University of the Philippines Manila, Manila 1000, Philippines; tjherbosa@up.edu.ph; 4 WHO Regional Office for South-East Asia, New Delhi 110002, India; buddhan@who.int; 5 The ASEAN Secretariat, Jakarta 12110, Indonesia; ferdinal.fernando@asean.org; 6 World Health Organization Centre for Health Development, Kobe 651-0073, Japan; kayanor@who.int

**Keywords:** health emergency and disaster risk management (H-EDRM), Sendai Framework for Disaster Risk Reduction, WHO Thematic Platform for H-EDRM, Emergency Medical Team, Emergency Medical Team Minimum Data Set, epidemiology, Public Health Surveillance

## Abstract

In October 2018, the World Health Organization (WHO) convened a meeting to identify key research needs, bringing together leading experts from WHO, WHO Thematic Platform for Health Emergency and Disaster Risk Management (Health-EDRM) Research Network (TPRN), World Association for Disaster and Emergency Medicine (WADEM), the Japan International Cooperation Agency (JICA), and delegates to the Asia Pacific Conference for Disaster Medicine (APCDM) 2018. The meeting identified key research needs in five major research areas for Health-EDRM. One of the five major research areas was “Health data collection during emergency and disaster”. Experts for this research area highlighted WHO Emergency Medical Team Minimum Data Set (EMT MDS), a standardized medical data collection method during and after disasters, as an example of substantial progress, with knowledge gaps and challenges in implementation in some regions and countries (i.e., information collection methodology in medical facilities of affected local areas, seamless and practical connection between acute phase data collection and post-acute phase local surveillance). The discussion on this research area also identified key research needs in standardization of broader health-related data to inform effective Health EDRM (i.e., community vulnerabilities, hospital functional status, infrastructure, lifelines and health workforce).

## 1. Introduction

In October 2018 at the Asia Pacific Conference for Disaster Medicine (APCDM) 2018 [1], an expert meeting to identify key research needs in major research areas was organized by the World Health Organization (WHO) Centre for Health Development (WHO Kobe Centre (WKC)), convening the leading experts from the Asia Pacific region, WHO, WHO Thematic Platform for Health Emergency and Disaster Risk Management (Health-EDRM) Research Network (TPRN), World Association for Disaster and Emergency Medicine (WADEM). The expert meeting was conducted, along with progress in the scientific aspect of the implementation of the Sendai Framework on Disaster Risk Reduction 2015–2030 [2], the resulting document of the 3rd UN World Conference on Disaster Risk Reduction (WCDRR), including establishment of TPRN, and following journal papers on recommended H-EDRM research activities [3,4,5,6]. Through the expert meeting and related review of literature and existing projects and activities, key research gaps in five major H-EDRM research areas were identified.

“Health Data collection before, during and after emergencies and disasters” is one of the five proposed major H-EDRM research areas. To conduct effective and timely health support in disaster relief and recovery activities, accurate and comprehensive health-related data is essential. Relief activities not based on relevant and accurate data could lead to negative impacts on recovery, related to the “do no harm” principle of Mary B. Anderson [7]. There have been a number of challenges in data collection in affected areas such as safe access to affected areas, preparing resources for data collection, obtaining informed consent from disaster victims, and fragmentation in data collecting and reporting methodology among different relief teams. All of these challenges cause a significant lack of scientific evidence in Health EDRM, especially the research with quantitative health data. For example, a MEDLINE search with MESH term “Disaster Medicine/statistics and numerical data” hit only 14 articles [8], which indicates a strong requirement of improvement in research and development of related methodology and tools for health data collection during and after disasters.

## 2. Material and Methods

The expert discussion of this research theme primarily aimed to identify recommended contents and source of health data to be collected, with reviewing the current available tools, methods and background systems and regulations in disaster relief activities. For the follow up of the expert meeting, a supplementary literature review and online discussion among the experts were conducted.

## 3. Results

Through the expert meeting based on a preliminary literature review, follow up online discussion, and a supplementary literature review, the experts developed the two research questions to be addressed, below. The greater details of the background of the proposed questions are described below.

(a) What are the national and regional challenges inhibiting implementation of the WHO standardized medical data collection systems during and after emergencies and disasters?
(b) What is the broader health-related data needed to inform effective Health-EDRM, i.e., community vulnerabilities, hospital functional status, infrastructure, lifelines and health workforce?

Firstly, the experts highlighted some key points for effective and practical data collection and utilization, including the “keep it simple” concept, which recommends simple and concise data, timely data collection for basic statistics in the manner to support operation, effective connection between collected data and ongoing response activities (i.e., recommendation to include geographical information).

Secondly, the current tools, methods and background systems and regulations in disaster relief were reviewed. A disaster is defined as a situation or event which overwhelms local capacity, necessitating a request for national or international level of assistance [9]. Although local health facilities are expected to function as a fundamental resource for disaster response, previous events revealed that local facilities often lose their functionally in major disasters. In a major disaster, a surge of affected patients further overwhelmed the damaged local capacities. To support local capacities by fulfilling the supply gaps, Emergency Medical Teams (EMTs), defined as a “groups of health professionals and supporting staff providing health care specifically to disaster- and health emergency-affected populations” are deployed to affected area [10]. To register as an EMT, verified by WHO, the team needs to meet EMT minimum standards, prepare resources for their activities by themselves, and regularly report their activities and status during their relief activities [11]. This nature of EMT supports the rationale for EMT to take a fundamental role for health data collection during and after disasters in collaboration with local capacities and under the agreement of affected countries. As EMTs can be deployed from different countries and organizations, the standardization of the data collection and reporting system has been a great challenge. Responding to this challenge, in 2017 EMT Minimum Data Set (MDS), a standardized medical data set to be collected and reported by EMT, was developed [12]. The fact that MDS became available was evaluated as an example of significant progress in health data collection by all of the experts who participated in the meeting.

Following the consensus on the improvement in health data collection by the development of MDS, the experts focused on the gaps and research needs for further comprehensive data collection to inform timely and effective disaster response and recovery. The expert meeting, and a following supplementary literature review, concluded that there is no internationally agreed or standardized methodology for public health data collection during and after a disaster, which indicates the substantial quality gaps between countries in post-acute phase health data collection and following response. The source and available tools and methods for health data collection are listed in Table 1.

Based on the proposed list of sources and tools for health data collection, several research priorities were identified. First, regarding the acute phase data collection, although WHO EMT MDS is available, some countries are not ready to implement it and need capacity-building and training to adapt their national system to meet international reporting standards. Experts supported research to analyze the best practices of and lessons from the implementation of MDS, for future effective capacity development in different countries.

Second, regarding data collection by and from local facilities, given the damaged capacity of local facilities during the acute phase, research on effective information collection and sharing systems through the collaboration between local facilities and EMTs is worth conducting. To address this research priority, including broader public health and environmental concerns (i.e., infrastructure, lifelines, hospital functional status, and health workforce available in communities) should be considered.

Third, in connection with the above-mentioned broader data collection, because there is no internationally agreed or standardized tool or method, research on setting up essential public health data for disaster response is expected to be conducted. The development process of WHO EMT MDS would inform the establishment of the broader data set.

Fourth, the experts also emphasized the critical knowledge gaps in an effective and harmonized transition from the acute phase, often relying on EMT activities to the post-acute phase relying on local capacities. Research on the seamless and effective connection of activities in those two phases is required.

## 4. Discussion

This expert discussion highlighted the needs and priorities in health data collection with a focus on on-site relief activities and medical support. To address the identified research needs for more comprehensive data for disaster response and recovery, inclusion of experts from other sectors such as United Nations International Strategy for Disaster Reduction, the World Bank and United Nations Development Programme will be the key. While the focus of this expert meeting was on post-disaster health data collection, understandably proper collection of baseline data before the disaster is essential to conduct efficient health emergency management.

In the Asia Pacific Region there are ongoing efforts to implement MDS, including the establishment and implementation of Surveillance in Post Extreme Emergencies and Disasters (SPEED) in the Philippines, and the Japanese version of SPEED (J-SPEED). Those two data collection systems have already been used for large scale data collection. For example, in Japan, the health data of 8089 patients in the Kumamoto Earthquake in 2016, 3620 patients in the West Japan Heavy Rain in 2018 and 591 patients in the Hokkaido Earthquake in 2018 were collected through the J-SPEED system, and are being analyzed quantitatively. This experience supports the practical utility of WHO EMT MDS. To maximize the impact of the data collection during and after a disaster, usually health data registration is required as the preparedness before disasters. Strengthening the health system, including capacity building for disaster response, is also the key for successful data collection. More implementation research on practical adaptation of international standards is also required, as countries which still need support for capacity building face the requirement of partial implementation of the MDS to address their ongoing health challenges in disasters.

## 5. Conclusions

The expert meeting on “Health data collection before, during and after emergencies and disasters” provided a clear view on the sources and currently available tools for health data collection during and after emergencies and disasters, and therefore required research activities for further improvement for comprehensive health data collection for effective, timely and seamless interventions in disaster response and recovery. More implementation research should be conducted.

## Figures and Tables

**Table 1:** Source and available tools or methods for health data collection during and after emergencies and disasters.

Source of Data (Reporter)	Acute Phase	Post-Acute Phase
Emergency Medical Team (EMT)	WHO EMT Minimum Data Set (MDS)	(Demobilization)
Local Facilities (i.e., Hospitals)	(Often difficult to function to collect data)	Local surveillance
Public Health Sector	(No international consensus)	Local surveillance
